# Cognitive speed–accuracy dissociation in multiple myeloma: a cross‑sectional study

**DOI:** 10.1186/s12868-026-01024-3

**Published:** 2026-07-10

**Authors:** Sumayyah Patel, Christopher Parrish, Frances Seymour, Melanie Burke

**Affiliations:** 1 School of Psychology, University of Leeds, Woodhouse, Leeds, LS2 9JT UK; 2 Leeds Institute of Clinical Trials Research, University of Leeds, Woodhouse, Leeds, LS2 9JT UK; 3 Leeds Teaching Hospitals, St James’ University Hospital, Beckett St, Leeds, LS9 7TF UK

**Keywords:** Blood cancer, Cognition, Processing speed, Drift diffusion modelling

## Abstract

**Purpose:**

This study examined whether adults with Multiple Myeloma (MM) show measurable cognitive differences compared with neurologically healthy controls. The primary research question focused on identifying domain-specific cognitive deficits and determining whether standard screening tools adequately capture cognitive abilities.

**Methods:**

A cross-sectional design compared 45 adults with MM to 40 controls. Participants completed 40–50 min of cognitive and psychological assessments, including the Montreal Cognitive Assessment (MoCA), validated measures of mood and daily functioning, and a digitised cognitive battery assessing key cognitive domains. Group differences in reaction time (RTs) and accuracy were analysed using ANCOVAs adjusting for age and education. Bayesian analyses and EZ drift diffusion modelling (EZ-DDM) were used to characterise evidence strength, prevalence of domain-level inefficiencies, and latent decision-making processes.

**Results:**

MoCA scores were lower in the MM group, although the group difference did not reach statistical significance. However, a higher proportion of MM participants met the criteria for possible cognitive impairment compared with controls. At the computerised task level, no significant group differences were observed in accuracy or mean RT. RT analyses showed significant effects of cognitive domain, age, and education, but no overall group effect or interaction. Despite this, threshold-based Bayesian analyses indicated that approximately 22% of MM participants exhibited multi-domain RT inefficiencies, with variability most evident in cognitive flexibility and semantic processing. Accuracy-based performance was largely preserved across both groups, with no participants exceeding impairment thresholds. EZ-DDM indicated that group differences were driven by reduced drift rates and prolonged non-decision times in MM, suggesting slower evidence accumulation with slower sensory encoding and/or motor execution.

**Conclusion:**

Cognitive differences in MM appear subtle and primarily expressed in processing efficiency rather than accuracy. Conventional screening measures and standard group comparisons may not fully capture this variability, particularly at the individual level. These findings support the use of more sensitive analytic approaches to characterise heterogeneous cognitive profiles in MM. Longitudinal and neuroimaging studies may help clarify whether observed inefficiencies reflect underlying neural changes associated with disease and treatment, and how these relate to functional outcomes.

**Supplementary Information:**

The online version contains supplementary material available at https://doi.org/10.1186/s12868-026-01024-3.

## Introduction

Cancer-Related Cognitive Impairment (CRCI) is an emerging, clinically neglected problem in non-CNS cancers, particularly in older adults. CRCI has been reported in multiple cancer populations, but its prevalence, mechanisms, and impact are not well documented. Multiple myeloma (MM) is an incurable plasma cell cancer accounting for 10% of haematological malignancies. Treatment advances have markedly improved survival rates; however, MM and the side-effects of therapy coupled with frequent comorbidities, polypharmacy and frailty, can exacerbate functional impairments [1]. The cognitive consequences of MM and its treatment on brain function remain largely underexplored and may not be well-managed in routine clinical practice. Given that MM disproportionately affects older adults, many of whom are already at risk of cognitive decline due to age or comorbidities, this gap poses direct challenges to patient-centred care, shared decision-making, and long-term survivorship planning.

Prior work in long-term survivors of haematologic malignancies including leukaemia, lymphoma, and MM has shown lasting cognitive impairments in executive functioning following bone marrow transplantation [2]. Executive dysfunction has been associated with poorer clinical outcomes and overall survival among patients receiving intensive treatment [3]. This underscores the prognostic value of cognitive screening and the need to integrate cognitive assessments into haematology care. CRCI appears multifactorial, driven by neuroinflammation from cytokine release and immune activation, oxidative stress from proteasome inhibitors and other MM therapies. Direct neurotoxic effects are evidenced by treatment-related structural and connectivity changes in the brain [4–6]. High-dose corticosteroids amplify these processes through hippocampal atrophy and impaired neurogenesis [7]. The chronic treatment-intensive nature of MM, combined with its prevalence in older adults, provides a useful model for examining the interplay of biological and therapeutic mechanisms underlying CRCI in non-CNS cancers.

While executive function is often used as an umbrella term, it encompasses multiple interrelated processes including cognitive flexibility, inhibitory control, working memory, and attentional regulation. These processes are supported by partially distinct neurocognitive mechanisms and may be differentially affected by ageing [8] and cancer-related factors [9]. While conceptually distinct, cognitive mechanisms such as processing speed are foundational to efficient executive functioning [10]. Slowed information processing is thought to reflect reduced neural efficiency, white matter integrity, and neurotransmitter dysregulation, and may precede measurable declines in accuracy on standard cognitive tests [11]. Reaction time (RT) provides a continuous, sensitive index of processing efficiency and attentional control, capturing subtle cognitive changes that may not be detected by accuracy-based alone [12]. This is relevant in MM where treatment-related neurotoxicity, fatigue, and systemic inflammation may disproportionately impact cognitive operations. Examination of both accuracy and RT allows for a more nuanced assessment of cognitive performance, distinguishing between task success and efficiency in achieving it.

A recent systematic review and meta-analysis by Patel et al. [13] found a significant decline in cognitive function, particularly within the first six months of MM treatment, compared with baseline. Unfortunately, contributing studies were largely based on limited self-reported data, highlighting the need for objective, controlled research measures. This underscores the need for systematically designed studies employing validated, computerised measures and well-characterised control groups to classify the cognitive profile of MM within the broader CRCI framework. While standard neuropsychological batteries are recommended in cancer research [14], research-based cognitive paradigms offer advantages in isolating specific cognitive processes and capturing continuous performance metrics such as RT, which may be more sensitive to subtle cognitive inefficiencies. The present approach therefore prioritised process-specific measurement over global composite scores.

The current study addresses this evidence gap by examining cognitive performance in adults with MM relative to neurologically healthy controls using a comprehensive, computerised cognitive testing battery based on established paradigms. The primary aim was to compare global cognitive function and domain-specific performance between MM patients and controls. Global cognition was assessed using the Montreal Cognitive Assessment (MoCA), while domain-specific performance was evaluated across tasks indexing working memory, attention, inhibition, semantic information processing, and flexibility. RT and accuracy were examined as complementary performance metrics across tasks, allowing differentiation between cognitive efficiency and task success. We hypothesised that MM patients would demonstrate slower RT, reflecting reduced processing efficiency, alongside potential differences in accuracy compared to controls. As a secondary aim, Bayesian modelling was used to estimate the prevalence and pattern of domain-specific inefficiencies within the MM group relative to controls. Finally, as an exploratory analysis, a summary-level EZ-Drift Diffusion Modelling (EZ-DDM) [15] approach examined latent decision parameters underlying observed response times and accuracy.

Methods

## Methods

### Design

This cross-sectional study was used to determine cognition in MM patients relative to healthy controls across the adult age span. Controls were recruited to achieve approximate comparability to the patient group in terms of age and educational background. Exact matching was not performed. The experiment was hosted in-person, using the online platform, Gorilla Experiment Builder (www.gorila.sc) [16].

Study conception was informed by self-reported cognitive concerns raised by patients and carers during routine clinical consultations, alongside input from clinicians involved in patient care. Although patients were not formally involved in the design, recruitment, or conduct of this study, these clinical observations helped shape the research focus and selection of outcome measures.

### Recruitment and ethical considerations

Patients were approached for recruitment using opportunity sampling during outpatient clinics at St James’ Hospital. Controls were recruited via caregiver roles and from the Older Adults Research Panel at the University of Leeds. Ethical approval was granted by the NHS Health Research Authority (REC Reference: 23/PR/0461, IRAS 325678; Granted: 16/06/23) and the University of Leeds Research Ethics Committee (PSYC- PSCETHS-782; Granted: 30/11/2023) (Supplementary Information 1). The study followed British Psychological Society [17] and Declaration of Helsinki guidelines [18]. Exclusion criteria were age < 18 years, usage of antipsychotic, antidepressant, antianxiety and antimanic medication, and known neurocognitive disorders (Alzheimer’s, Parkinson’s). Patient eligibility was confirmed via medical record review by healthcare professionals.

### Measures

Testing took ~ 40–50 min in quiet controlled environments under research supervision on a standardised laptop. Demographic and clinical data were collected using self-report and NHS medical records, including age, gender, nationality, social status, education, language proficiency, sleep duration, time since diagnosis, and treatment profile (steroid use and transplant status).

Self-reported measures included the Patient Health Questionnaire-9 (PHQ-9) [19], a 9-item diagnostic screening tool for the presence and severity of depression, and the Generalised Anxiety Disorder assessment-7 (GAD-7) [20], assessing anxiety severity. Functional independence was evaluated using the Activities of Daily Living (ADL) scales, comprised of two subscales including Lawton-Brody’s Instrumental ADL [21] and Katz Index of Independence [22], which assess more complex functionality (like managing finances, shopping, food preparation, transportation) and basic daily functioning (like bathing, dressing, toileting), respectively. Descriptions and scoring details of all self-report measures are provided in Supplementary Information 2 A.

All participants completed a pen-and-paper Montreal Cognitive Assessment (MoCA) [23], a standardised global cognitive tool sensitive for detecting Mild Cognitive Impairment (MCI) and early dementia, well-validated in CRCI contexts [24, 25].

Domain-specific cognitive performance was assessed using a computerised battery. Tasks were based on established experimental paradigms targeting visuospatial working memory, cognitive flexibility, depth of processing, attention, and inhibitory control. Key task features are summarised below, with full procedural details (stimuli, timing, and scoring) provided in Supplementary Information 2B.

(i) *Working Memory*.

A computerised version of the Corsi block-tapping test assessing visuospatial working memory [26], described in detail and validated by Kessel et al. [27] and in clinical populations by Siddi et al. [28] in which participants reproduced sequences of spatial locations presented on-screen across multiple trials. Each trial included five locations. Participants completed 8 trials in total including one practice trial.

(ii) *Cognitive Flexibility*.

A cued task-switching paradigm assessing cognitive flexibility [29], adapted from Ravizza and Carter [30], requiring participants to alternate between colour- and shape-based decision rules on a trial-by-trial basis. This task has been validated previously for psychometric testing of executive function with good reliability (ICC ≥ 0.68) [31]. For 16 trials, participants responded to cue words (‘shape’ or colour’) by clicking either the ‘F’ key if the object was blue or square, or ‘J’ if it was green or a rectangle.

(iii) *Levels of processing task*.

An assessment of the depth of semantic encoding [32] in which participants made structural, phonemic, or semantic judgements by selecting ‘Yes’ or ‘No’ on the screen. Following 30 trials, a delayed recall phase required participants to type as many items as possible. For the primary analysis, accuracy was defined as the proportion of correct Yes/No responses during the encoding phase, reflecting processing efficiency at different levels of semantic depth. The recall phase was included to support the encoding manipulation but was not used as the primary outcome measure. The Levels of Processing task has strong experimental construct validity, demonstrated by systematic and replicable effects of orienting task on later memory performance [33]. As the paradigm is designed as an encoding manipulation rather than a trait measure, traditional psychometric indices such as internal consistency and test–retest reliability are not applicable to condition contrasts.

(iv) *Posner cueing task*.

A digital assessment of attention [34, 35]; where participants responded to spatial targets following valid or invalid cues. Participants responded with the ‘F’ key if the target stimulus appeared in the left box and ‘J’ when on the right. In several trials, a green circle (cue) appeared either congruently or incongruently to the target stimulus. There were 22 trials in total including two practice trials. This task has been widely used and validated in both control and clinical populations for assessing spatial orientation of attention [36].

(v) *Stop-signal delay task.*.

An assessment of inhibitory processing [37], as detailed in Verbruggen et al. [38] requiring participants to respond to directional stimuli while withholding responses on a subset of trials. Participants must respond by pressing the ‘F’ or ‘J’ key to acknowledge whether the arrow is pointing to the left or right. Two practice trials were provided. This task has been validated and demonstrates moderate to good test–retest reliability across healthy and clinical populations, including computerized implementations [39].

### Procedural control and data quality

Many of these paradigms have demonstrated sensitivity to cognitive processes in prior cancer research [40, 41]. Tasks were implemented in a computerised format to enable standardised administration across participants without modification to core cognitive demands. Task structure, stimulus properties, and response requirements were preserved relative to the original validated paradigms, with adaptation limited to digital delivery. All assessments were conducted in controlled environments using identical equipment and standardised instructions across participants.

All cognitive assessments were administered by trained research assistants under the direct supervision of the lead investigator, SP. Testing procedures were identical for patient and control groups, with all participants receiving standardized task instructions prior to each task.

To minimise confounds, testing was conducted in quiet environments with minimal external distractions. For hospital-based assessments, dedicated clinical rooms were used to reduce interruptions. Participants were monitored throughout testing to ensure task comprehension and monitor engagement and task adherence where possible. The online experimental platform provided automated logging of response accuracy, timing, and task integrity, allowing identification of corrupt or incomplete datasets, which were excluded from analysis. Trials were excluded where there was clear evidence of non-compliance due to technical interruption.

### Data analysis

Statistical analyses were conducted using R Studio 2025.05.0. Corrupted datasets (*N* = 3) were excluded prior to analysis. Missing data was minimal and attributable to data handling issues rather than non-response. Age data were missing for two control participants. MoCA scores were missing for seven participants, all within the MM group, due to administrative data loss. Given the low proportion of missing data (< 10%) and the absence of systematic patterns, group-wise mean imputation was applied; education was not imputed. Sensitivity analyses comparing imputated and complete-case results yielded comparable results, indicating the imputation approach did not materially affect findings.

Structural, phonemic, and semantic processing were analysed both individually and as a composite Levels of Processing (LoP) score, to evaluate both global and process-specific effects. Subdomain scores were included in all analyses.

Group differences in MoCA scores, psychological wellbeing and daily functioning were assessed using Wilcoxon rank-sum tests. Cognitive performance was analysed using two separate two-way ANCOVAs: (i) mean RT (ms), and (ii) mean accuracy scores (%), with age and education as covariates. RTs were analysed within-task and were not standardised or compared across paradigms, given differences in task structure and baseline response demands. Sex was not included as a covariate due to model stability considerations and sample size constraints. Although some assumptions (normality and homogeneity of variance) were violated, relationships with covariates were approximately linear and given the moderate sample size (*N* = 85) and robustness of ANCOVA, results are interpreted cautiously. Adjusted means and effect sizes are reported.

Bayesian statistical framework was conducted in parallel to quantify the strength of evidence for group differences. Weakly informative normal priors centered at zero were specified, with model diagnostics confirming convergence. Results are reported as posterior means with 95% credible intervals (CrI) and Bayes factors (Supplementary Information 3).

Individual-level impairment was also characterised using a normative threshold approach, defined as performance ≥ 1.5 standard deviations from the control group mean consistent with CRCI guidelines [14, 42]. Prevalence rates were calculated as the proportion of MM participants exceeding these thresholds across domains.

These analytic approaches were selected to address complementary questions. ANCOVA evaluated covariate-adjusted group differences at the mean level, Bayesian analyses quantified the strength of evidence for these effects, and threshold-based analyses characterised individual-level variability relative to the control distribution. Threshold-based classifications are interpreted as descriptive rather than inferential.

EZ-DDM was applied to summary-level RT and accuracy data to explore latent decision-making parameters: drift rate (v), boundary separation (a), and non-decision time (Ter), indexing evidence accumulation, response caution, and sensory–motor processes, respectively. Parameters were estimated at the participant-level for each domain and summarised descriptively at the group level. EZ-DDM was used to support interpretation rather than to derive precise mechanistic estimates.

## Results

Data from 50 patients with MM and 43 neurologically healthy controls with normal or corrected-to-normal vision were collected. Three participants withdrew during testing due to task frustration, computer discomfort, or clinical distress. The final sample included 45 MM participants and 40 controls. Participant demographics are summarised in Table 1.

**Table 1:** Summary Statistics of Participant Characteristics

Variables	MM	Controls
Demographics		
*N* (*%*)	45 (52.94)	40 (47.06)
*Age*,* Mean (Range)*	65.75 (33.83–90.33)	69.49 (35.17–85.58)
> 80y *N*(*%*)	4 (9)	2 (5)
*Sex*,* N(%)*		
Male	34 (75.6)	13 (33.3)
Female	11 (24.4)	26 (66.6)
*Nationality*,* N*		
White, non-Hispanic	44	40
Black	1	0
*Education*,		
Mean yrs (SD)	13.2 (1.69)	15 (2.49)
Prefer not to say, *N* (*%*)	5 (11.1)	7 (17.5)
**Questionnaire(s)**, ***M*(*SD*)**		
MoCA	25.4 (2.67)	26.33 (2.54)
MCI, *N (%)*	23 (51.1)	11 (27.5)
PHQ-9	7.7 (5.04)	10.8 (8.34)
GAD-7	4.6 (4.11)	6.6 (6.16)
*Activities of Daily Living (ADL)*		
Lawton-Brody Instrumental ADL	6.9 (1.60)	7.88 (0.82)
Katz Index	5.6 (0.80)	5.96 (0.16)
**Sleep**, ***N(%)***		
Good	14 (31.11)	15 (37.5)
Moderate	26 (57.78)	25 (62.5)
Poor	5 (11.11)	0 (0)
**Treatment**		
On steroids *N (%)*	31 (69)	NA
*Unknown* ^*a*^	4 (9)	NA
HSCT *N* (*%*)	25 (56)	NA
Transplant in-eligible	13 (29)	NA

Note. MM = Multiple Myeloma; MoCA = Montreal Cognitive Assessment; MCI = Mild Cognitive Impairment; PHQ-9 = Patient Health Questionnaire 9; GAD-7 = Generalised Anxiety Disorder 7; ADL = Activities of Daily Living; HSCT = Haematopoietic Stem Cell Transplantation; NA = Not Applicable

^a^Patients with unknown steroid treatment status were auto-referrals to the hospital; treatment data were unavailable for these cases

- *Cognitive and Psychosocial Screening*.

***Montreal Cognitive Assessment (MoCA)***.

Group difference did not reach statistical significance (*W* = 1105, *p*=.070), though MM participants scored lower (*M* = 25.4, *SD* = 2.67) than controls (*M* = 26.3, *SD* = 2.54). Classification based on the established clinical cutoff, indicated that 51.1% of MM participants and 27.5% of controls fell below the threshold for potential MCI.

***Depression and Anxiety (PHQ-9 and GAD-7)***.

Moderate depressive symptoms (PHQ-9) were reported by 13% of MM participants (6/45) and 28% of controls (15/40); more severe depression was rare in both groups. Moderate anxiety symptoms (GAD-7) were reported by 13% MM participants (6/45) versus 20% of controls (8/40), with severe anxiety observed only in controls (10%, 4/40). No group differences were statistically significant in depression (PHQ-9: *W* = 1086, *p* = .101) or anxiety (GAD-7: *W* = 978, *p* = .490).

***Daily functioning (ADLs)***.

MM participants showed significantly lower functioning than controls on both instrumental (Lawton *W* = 1302, *p* < .001:) and basic ADLs (Katz: *W* = 1079.5, *p* = .0068).

***Sleep quality***.

Self-reported sleep quality was broadly comparable between groups (MM: Good = 14, Moderate = 26, Poor = 5; Controls: Good = 15, Moderate = 25, Poor = 0).

b. *Mean Reaction Time (RT)*.

Factorial ANCOVA revealed no significant effect of group on RT (*F*_(1, 566)_ = 1.87, *p* = .172). There was a significant effect of cognitive domain (*F*_(7, 566)_ = 13.61, *p* < .001), and among covariates of age (*F*_(1, 566)_ = 8.74, *p* = .003) and years of education (*F*_(1, 566)_ = 10.97, *p* = .001) on RT. There was no significant group x cognitive domain interaction (*F*_(7, 566)_ = 0.17, *p* = .99).

**Fig. 1:**
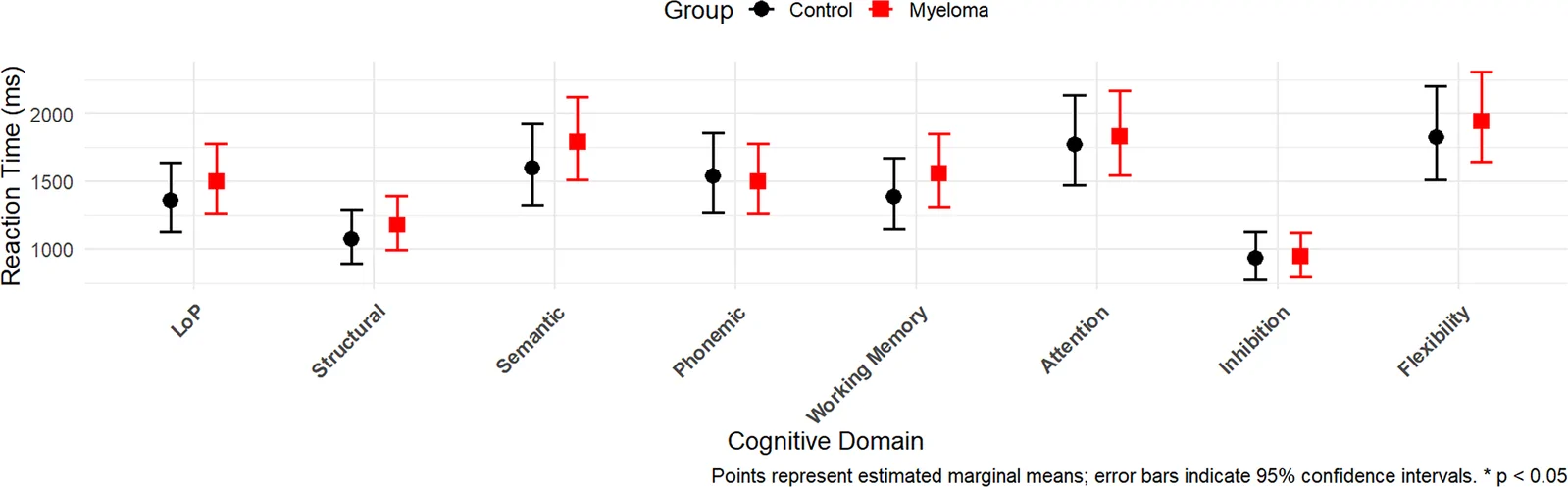
Estimated marginal mean reaction times (ms) across cognitive domains in myeloma patients and controls, adjusted for age and years of education. Error bars indicate 95% confidence intervals. Note *LoP = Levels of Processing - with structural*,* semantic*,* and phonemic being subsets of this overall score*

Estimated marginal means (EMMs), adjusted for age and years of education, indicated comparable RTs between groups and across domains. Although MM participants exhibited slower RTs than controls, differences were small and did not reach significance, with Hedges’ g values ranging from − 0.218 to 0.047 (all *p* > .35). EMM RTs are presented in Fig. 1, and linear trends by age and education within each group and cognitive domain are illustrated in Supplementary Information 4.

Threshold-based prevalence analysis revealed the highest RT impairment in cognitive flexibility (17.8%, *N* = 8), followed by semantic processing (13.3%, *N* = 6), LoP (11.1%, *N* = 5), working memory (11.1%, *N* = 5), and inhibition (11.1%, *N* = 5). Attention (6.67%, *N* = 3) and structural processing (4.44%, *N* = 2) showed lower impairment, and phonemic processing showed no impairment. Further analysis indicated 22.2% of MM patients had deficiencies in ≥ 2 domains (*N* = 10), but only 11.1% in ≥ 3 cognitive domains (*N* = 5). Multi-domain RT threshold exceedance was primarily observed in semantic processing and cognitive flexibility, followed by LoP, inhibition, and working memory. To contextualise threshold-based classification, the proportion of control participants exceeding the ≥ 1.5 SD criterion (derived from the control distribution) was also computed. Across domains, this ranged from 2.5 to 10%, consistent with expected tail probabilities under a standard deviation-based cut-off.

c. *Mean accuracy*.

**Fig. 2:**
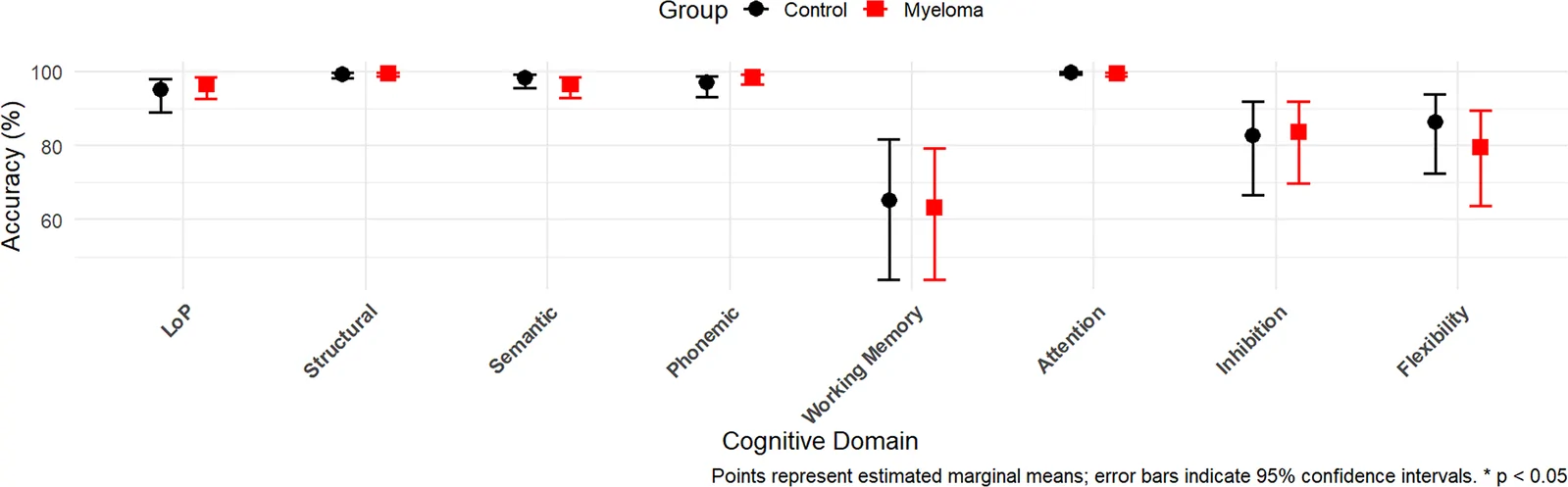
Estimated marginal mean accuracy (%) across cognitive domains in myeloma patients and controls, adjusted for age and years of education. Error bars indicate 95% confidence intervals. *Note: LoP = Levels of Processing - with structural*,* semantic*,* and phonemic being subsets of this overall score*

Accuracy revealed no significant group effect (*F*_(1, 566)_ = 0.006, *p* = .939). There were significant effects of cognitive domain (*F*_(7, 566)_ = 32.52, *p* < .001), age (*F*_(1, 566)_ = 10.71, *p* = .001) and years of education (*F*_(1, 566)_ = 25.95, *p* < .001). The group x cognitive domain interaction was not significant (*F*_(7, 566)_ = 0.59, *p* = .767). EMMs revealed accuracy was generally high across all domains for both groups, with mean percentages ranging 63–99% depending on the task (Fig. 2) and Hedges’ g values ranging from − 0.258 to 0.235 (all *p* > .27).

Accuracy-based performance was consistently high across domains in both groups, with no participants exceeding the ≥ 1.5 SD threshold in any domain. Consequently, threshold-based classification revealed accuracy-based performance was largely preserved with no domain showing measurable impairments.

d. EZ-Drift-Diffusion Modelling (EZ-DDM).

**Fig. 3:**
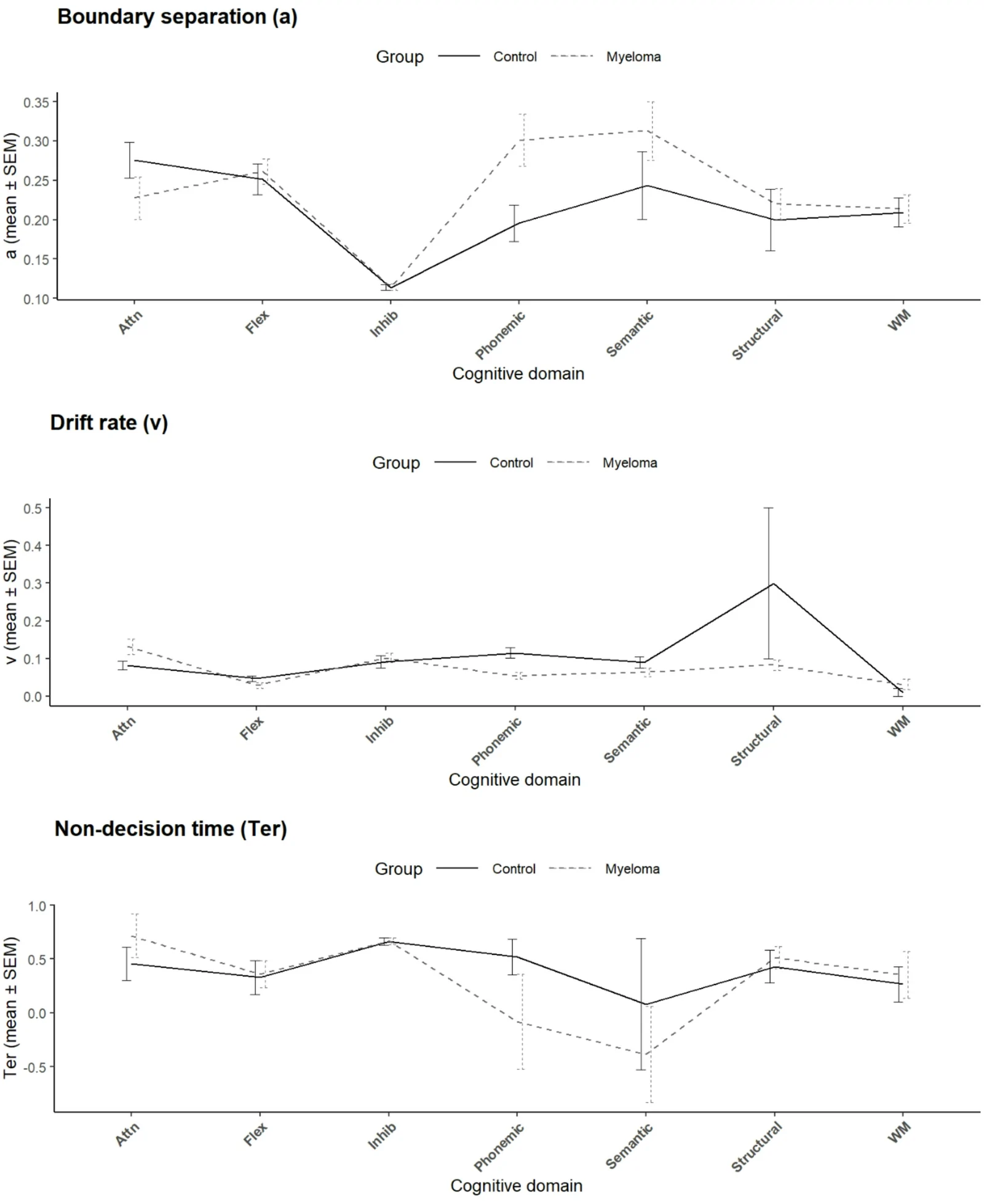
EZ-DDM parameter estimates (boundary separation, drift rate, non-decision time) across cognitive domains. Solid lines indicate control participant responses and dashed lines individuals with myeloma. Error bars indication standard error of the mean. Attn = Attention; Flex = Flexibility; Inhib = Inhibition; WM = Working Memory

EZ-DDM estimated latent decision-making parameters for each cognitive domain. Group means (± SD) for boundary separation (a), drift rate (v), and non-decision time (Ter) are visualised in Fig. 3. EZ-DDM parameters were estimated only for participant–domain combinations with non-degenerate accuracy; exclusions were applied at the domain level only.

Boundary separation was broadly comparable between groups, indicating similar response caution (Control: a_mean range = 0.006–0.019; MM: a_mean range = 0.006–0.016). Drift rate was generally lower in MM participants than controls across most domains (e.g., phonemic: 3.79 vs. 1.89; semantic: 2.68 vs. 1.83) except for attention, where rates were higher in MM than controls. However, attention accuracy was near ceiling in the full sample, resulting in a disproportionate exclusion of control participants when applying the non-degenerate accuracy criterion (0 < PC < 1). Consequently, the attention drift-rate comparison is based on a smaller and potentially unrepresentative subset (Control: *N* = 12; MM: *N* = 17) and should be interpreted cautiously.

Non-decision time (Ter), reflecting sensory encoding and motor execution, was typically longer in MM participants (e.g., phonemic: 1.51 vs. 2.32; semantic: 1.98 vs. 2.59), suggesting modest delays in pre- and post-decision processing.

## Discussion

Our research assessed cognition in adults with MM relative to controls using global screening and domain-specific computerised tasks. Over half of MM participants met the MoCA threshold for potential MCI, compared with less than a third of controls. No significant group differences were observed in depression or anxiety symptoms. However, MM participants showed reduced functional independence on both instrumental and basic activities of daily living. At task level, group differences in RT and accuracy did not reach statistical significance, although Bayesian analyses indicated variability in performance across cognitive domains, particularly in processing speed-related measures.

Specifically, 51% of the patients with MM met the criteria for MCI on the MoCA [23] compared to 27.5% of controls. While the former findings are consistent with previous research on objective cognitive deficits in haematological malignancies [2, 3], dichotomisation of MoCA scores using clinical thresholds may overstate group differences in the absence of significant continuous effects, thus classifications should be interpreted as indicative rather than diagnostic. Notably, the prevalence in controls exceeds the expected ~ 15% MCI in community-dwelling adults [42]. This likely reflects sample characteristics, as over one-third (37.5%) were relatives or carers attending outpatient appointments. Carers also reported high rates of depressive and anxiety symptoms (93% and 33% respectively) compared to MM participants (13% for both). Depression is known to impair cognitive performance [43], so the inflated MCI prevalence in controls may highlight caregiver burden.

Functional independence was reduced in participants with MM on the Katz and Lawton scales, indicating some loss of autonomy even in routine daily tasks. This aligns with extensive evidence from cognitive ageing literature demonstrating robust associations between global cognition, executive function, and IADL performance, particularly in those over 60 years of age [44, 45]. Declines in domains like attention, working memory, and executive control have been consistently linked to reduced capacity for complex daily activities including medication management, and financial decision-making [46], . In wider CRCI contexts, previous studies report associations between broader executive function and functional outcomes [47], with processing speed often identified as one contributing factor [48]. Accordingly, while processing speed showed the most consistent signal across analyses, functional outcomes are likely influenced by a broader interaction of attentional control, working memory, and executive efficiency.

At the domain-specific level, digitalised cognitive testing revealed performance differences between groups that were not captured by null-hypothesis testing alone. While ANCOVAs showed no significant group effects, Bayesian prevalence modelling identified meaningful variability in RT-based measures, particularly within semantic information processing and cognitive flexibility tasks. These findings should be interpreted as indicative of potential cognitive inefficiencies rather than definitive impairments, particularly given the exploratory nature of the analytic approach and the lack of formal psychometric validation of the adapted task versions in MM populations.

These observations align with prior studies showing subtle domain-specific deficits in executive functions during chemotherapy, influenced by biological and demographic factors [49]. Though causal inference regarding underlying mechanisms cannot be applied definitively, processing speed deficits may relate to white matter changes and neurotransmitter dysregulation [50]. Nearly half (49%) our MM cohort underwent Heamatopoietic stem cell transplantation, a regimen involving high-dose chemotherapy and immunosuppressive agents, alongside corticosteroid exposure (64%), both of which have been previously associated with neurobiological effects, including potential impacts on white matter integrity [51–52] (Supplementary Information 5). However, no consistent reduction in MoCA scores was observed in participants with HSCT or steroid exposure; if anything, MoCA scores were marginally higher in these subgroups, suggesting that any treatment-related cognitive effects may be subtle or domain-specific and not detectable using global cognitive screening measures.

Our findings also suggest reliance on group accuracy averages in cognitive tests may underestimate CRCIs in MM and potentially other patients. Threshold-based modelling showed cognitive flexibility was particularly affected, and over 20% of MM participants exceeded the ≥ 1.5 SD threshold revealing potential processing speed inefficiencies in two or more cognitive domains, whereas fewer individuals met criteria for more extensive multi-domain exceedance. RT-based measures captured greater heterogeneity in performance than accuracy-based outcomes, suggesting that a subset of individuals may show reduced efficiency despite non-significant group-level effects.

Exploratory EZ-DDM analyses were used to identify mechanisms underlying slowed processing. While boundary separation (response caution) was broadly comparable between groups, drift rate (evidence accumulation) and non-decision time typically took longer in MM participants. This pattern is broadly consistent with slower information processing, reduced efficiency in decision-making, and modest delays in sensory encoding and motor response execution. This supports the view that slower processing is driven by reduced evidence accumulation rather than changes in response caution [15]. These estimates should be interpreted cautiously given potential parameter instability associated with small sample sizes and the simplified model structure.

Therapies managing cancer such as chemotherapy, immunotherapy and corticosteroids may contribute to poor processing speed in MM [48, 53]. Impaired myelination and altered neurotransmitter signalling may additionally contribute to subtle neural connectivity changes, providing a potential avenue for future interventions [54]. Given expanding MM therapeutic options and their effect on quality of life, neurocognitive sequelae of MM therapies warrant closer scrutiny. Evidence increasingly indicates corticosteroids are a likely culprit, and high-dose prolonged use should be carefully considered in MM and other chronic diseases [55–57].

Several limitations should be acknowledged. First, the cross-sectional design precludes inference about cognitive trajectories or causal relationships between treatment and cognition. Second, potential self-selection bias from outpatient-only recruitment, whereby individuals receiving more intensive therapies and/or with more severe cognitive impairment may have been unable to participate. Similarly, although controls were recruited to be comparable to patients, slight differences in age, education, and gender distribution remained. These factors are known to influence cognitive performance and were therefore included as covariates where appropriate; however, residual confounding cannot be fully excluded. Sleep quality and fatigue were not formally modelled in the current analyses but may represent additional factors influencing attention and processing speed. Although overall distributions were broadly similar between groups, a small proportion of MM participants reported poorer sleep, which may have contributed to variability in cognitive performance. Additionally, heterogeneous treatment regimens limited isolation of the independent therapeutic effects, or dissection of such effects from disease-driven changes, both of which warrant further investigation. Finally, several tasks reached ceiling accuracy (particularly attention, structural, and phonemic processing), suggesting some tests were insufficiently challenging although working memory, inhibition, and cognitive flexibility did not reach ceiling and still showed no significant group differences.

A key strength of this study is the integration of objective cognitive measures with functional outcomes, allowing exploration of how subtle cognitive variability relates to everyday functioning. This combined approach provides ecologically relevant insight into how cognitive processes may translate into real-world functioning, which remains underexplored in MM. the inclusion of a comparative control group further supports interpretation by enabling differentiation of CRCI from typical ageing effects.

The computerised cognitive tasks were derived from established experimental paradigms; however, as noted, the adapted versions used in this study have construct validity, but have not undergone formal psychometric validation in MM populations. Accordingly, findings derived from these measures should be interpreted as exploratory indicators of cognitive processes rather than definitive neuropsychological assessments in MM.

MM serves as a useful exemplar for studying CRCI due to its chronic, treatment-intensive course. Similar cognitive effects are likely to occur across other haematological malignancies [58], highlighting broader clinical applications and potential challenges. Improved understanding of the neurocognitive mechanisms underlying CRCI could inform treatment selection and strategies to protect cognitive function. Future research should prioritise longitudinal designs to track cognitive change over time alongside multimodal neuroimaging approaches like functional magnetic resonance imaging (fMRI) and functional near-infrared spectroscopy (fNIRS) [59–60] to better characterise underlying neural mechanisms. This may help disentangle age-, disease-, and treatment-related contributions to cognitive variability and identify vulnerable subgroups most at risk of functional decline.

## Conclusions

While overall cognitive accuracy was relatively well-preserved in adults with MM, subtle differences in processing efficiency may be present when more sensitive, domain-specific measures are applied. Rather than reflecting uniform impairment, cognitive performance in MM appears heterogeneous, with individual differences likely influenced by a combination of disease-, treatment-, and demographic-related factors. Patterns observed in evidence accumulation and decision-making processes are consistent with reduced cognitive efficiency. Our results underscore the importance of sensitive, domain-specific cognitive assessments in routine cancer care and suggest that testing of processing speed may highlight vulnerable subgroups in a way that may not be captured by conventional screening tools alone. However, present findings are not sufficient to support definitive mechanistic conclusions. Future research should investigate longitudinal and mechanistic studies to clarify trajectories of cognitive changes and disentangle the relative contributions of disease progression, treatment exposure, and ageing. Such work may inform the development of targeted interventions aimed at enhancing neuroplasticity and mitigating cognitive decline in this population.

## Supplementary Information

Below is the link to the electronic supplementary material.

Supplementary Material 1

## Data Availability

De-identified participant data supporting the findings of this study are available from the corresponding author upon reasonable request. Requests should include a brief description of the intended use of the data.
