# Supplementary material for: Cognitive speed–accuracy dissociation in multiple myeloma: a cross‑sectional study
Source: BMC Neurosci. 2026 Jul 10;27:41. doi: 10.1186/s12868-026-01024-3 (PMC13640288; doi:10.1186/s12868-026-01024-3)
Supplement: Supplementary file 1 [file 12868_2026_1024_MOESM1_ESM.docx]

***Title:***
Cognitive Speed–Accuracy Dissociation in Multiple Myeloma: A Cross‑Sectional Study

***Journal:***
BMC Neuroscience

***Author(s):***
**Sumayyah Patel, BSc**
University of Leeds, Woodhouse, Leeds LS2 9JT, UK
ORCID: 0009-0006-7717-5548
Email: [ps19sp@leeds.ac.uk](mailto:ps19sp@leeds.ac.uk)

**Christopher Parrish,** **MBChB,** **PhD**
University of Leeds, Woodhouse, Leeds LS2 9JT, UK
Leeds Teaching Hospitals, St James’ University Hospital, Beckett St, Leeds LS9 7TF, UK

**Frances Seymour, MBChB, PhD**
Leeds Teaching Hospitals, St James’ University Hospital, Beckett St, Leeds LS9 7TF, UK

**Melanie Burke,** **PhD**
University of Leeds, Woodhouse, Leeds LS2 9JT, UK

**SUPPLEMENTARY INFORMATION 1
Patient** **Participant Information Sheet**

**Title of study:** Characterising cognitive function in multiple myeloma

**Researcher:**	Dr Christopher Parrish

**Address:**	Department of Haematology

Leeds Teaching Hospitals NHS Trust

Beckett Street Leeds

LS9 7TF

**Sponsor:**	Leeds Teaching Hospitals NHS Trust

Beckett Street, Leeds

LS9 7TF

**Ethics reference:** 23/PR/0461

**You are being invited to take part in a research study.**

Before you decide whether to take part it is important for you to understand why the research is being done and what it will involve. Please take time to read the following information carefully. Part 1 tells you about the study and what is involved. Part 2 tells you how we will use and protect your personal data. Please ask us if there is anything that is not clear or if you would like more information. Take time to decide whether you wish to take part.

**PART 1**

**What is the purpose of the study?**

We want to understand whether having multiple myeloma and treatment for myeloma has an impact on the way the brain processes and uses information (we call this cognitive function).

**Why have I been invited?**

You have been invited to take part because you have a diagnosis of multiple myeloma. Cognitive function can change as we get older, or with other factors, so as well as patients with myeloma we are also approaching some people of a similar age without myeloma so that we can tell whether any specific changes occur due to multiple myeloma.

Subjects who wish to take part in this study will be asked whether they are known to have any conditions which might affect the results (for example conditions such as Alzheimer’s disease or Parkinson’s disease); if you wish to proceed the study team will complete a checklist with you before starting to ensure you are suitable to take part.

**Do I have to take part?**

No. It is up to you to decide whether to take part. If you do, you will be given this information sheet to keep and be asked to sign a consent form. You are still free to withdraw at any time during the duration of the study and without giving a reason. A decision to withdraw at any time, or a decision not to take part, will not affect the standard of care you receive. If you chose to withdraw during the study we will use the data we have collected up until the point that you withdraw but we will not collect any further information about you.

**What do I have to do?**

If you agree to take part you will be asked to sign a consent form. We will then ask you to complete a short questionnaire about your physical and mental health, and day-to-day activities, which will take around 5-10 minutes. We are asking these questions in case other health problems effect cognitive function. Your answers to these questions will not affect the care you receive for your myeloma. If you have questions about any of the health aspects covered by the questionnaire, you should discuss this with the team treating your myeloma.

You will then be asked to complete a set of tests on a computer, which will measure the way your brain processes and uses information and will take approximately 30 minutes. Some people who take part will be asked to wear a head cap containing some light emitting diodes (LEDs) and detectors while they perform these tasks. This won’t restrict the movement of your head or body and shouldn’t be uncomfortable. This will measure how blood and oxygen flow to different part of the brain as you process and use information.

If you attend the clinic again during the time the study is running you will be invited to complete the health questionnaire and cognitive tests again but we will not ask you to complete the cognitive tests more often than once per month. The study will run for 3 years and we may invite you to complete the tests more than once during this time. On each occasion you always have the choice about whether you wish to repeat the tests.

We will also collect some routine information from your medical record such as information from blood test results and scans. We will use a unique study code to share this information with the study team so it will not be linked to your personal details like name or date of birth.

**What are the possible benefits of taking part?**

The information we collect might help improve the treatment and monitoring of people with multiple myeloma.

**What are the possible disadvantages of taking part?**

The risks from taking part in this study are minimal., but it will require some of your time, for which we are very grateful. We will try to ensure the cognitive tests are done whilst you are already waiting in clinic whenever possible. All study activities are covered by the NHS indemnity scheme.

**Are there any restrictions on who can take part?**

You must be over 18 years of age to take part. If you have an existing health condition that affects cognitive function, such as Alzheimer’s disease, you will not be able to take part. Please discuss this with the research team if you are unsure.

**What will happen to any samples I give?**

We will not be collecting any samples from you during this study.

**What will happen to the results of this study?**

The results will be published in a university report and may be reported in peer-reviewed journals and presented at meetings. You will not be identified in any reports.

**Who is organising the study?**

The study is being organised by Dr Christopher Parrish from the Department of Haematology at Leeds Teaching Hospital and Dr Melanie Burke from the School of Psychology at the University of Leeds. The cognitive tests will be run by Miss Sumayyah Patel, who is a PhD student from the University of Leeds and the results will form part of her PhD thesis.

**Who has reviewed the study?**

All research in the NHS is looked at by an independent group of people called a Research Ethics Committee. A favourable ethical opinion has been obtained [REC: 23/PR/0461; IRAS ID: 325678; Version 14.0].

**Who should I contact if I have a complaint?**

If you wish to make a complaint about this study or have been harmed as a consequence of this study please contact the PALS service on 0113 2066261. textphone: 07468753025 (if you are D/deaf or speech impaired) email: patientexperience.leedsth@nhs.net.

**PART 2**

**How will we use information about you?**

We will need to use information from you for this research project. We will also use information from your medical record. This information will include your name, NHS number and date of birth.  People will use this information to do the research or to check your records to make sure that the research is being done properly. People who do not need to know who you are will not be able to see your name or contact details. Your data will have a code number instead. We will keep all information about you safe and secure.

Once we have finished the study, we will keep some of the data so we can check the results. We will write our reports in a way that no-one can work out that you took part in the study.

**What are your choices about how your information is used?**

- You can stop being part of the study at any time, without giving a reason, but we will keep information about you that we already have.
- We need to manage your records in specific ways for the research to be reliable. This means that we won’t be able to let you see or change the data we hold about you.

**Where can you find out more about how your information is used?**

You can find out more about how we use your information

- at [www.hra.nhs.uk/information-about-patients/](https://www.hra.nhs.uk/information-about-patients/)
- our information on our website <https://www.leedsth.nhs.uk/patients-visitors/patient-and-visitor-information/how-we-use-your-data/>
- by asking one of the research team
- by sending an email to [Leedsth-tr.informationgovernance@nhs.net](mailto:Leedsth-tr.informationgovernance@nhs.net)
- by ringing us on 0113 2433144 and ask for the Data Protection Officer

**IRAS ID: 325678**

**Centre:** Leeds Teaching Hospital NHS Trust

**Participant Identification Number for this trial:**

**CONSENT FORM**

**Title of Project:** Characterising cognitive function in multiple myeloma

**Name of Researcher:** Dr Christopher Parrish

Please initial box

1. I confirm that I have read the information sheet dated.................... (version............) for the above study. I have had the opportunity to consider the information, ask questions and have had these answered satisfactorily.
2. I understand that my participation is voluntary and that I am free to withdraw at any time without giving any reason, without my medical care or legal rights being affected.
3. I understand that relevant sections of my medical notes and data collected during the study, may be looked at by individuals from regulatory authorities or from the NHS Trust, where it is relevant to my taking part in this research. I give permission for these individuals to have access to my records.
4. I understand that the information collected about memay be used to support other research in the future, and that information may be shared anonymously with other researchers.
5. I agree to take part in the above study.
6. OPTIONAL: I also consent to taking part in the optional study where my brain patterns will be recorded by wearing a special light-producing cap.

Yes:

No:

Name of Participant	Date	Signature

Name of Person	Date	Signature

taking consent

1 copy to be retained by the research team within the study site file

1 copy to be provided to the participant

1 copy to be entered into the participant’s medical records

**Control Participant Information Sheet**

**Title of study:** Characterising cognitive function in multiple myeloma

**Researcher:**	Dr Christopher Parrish

**Address:**	Department of Haematology

Leeds Teaching Hospitals NHS Trust

Beckett Street Leeds

LS9 7TF

**Sponsor:**	Leeds Teaching Hospitals NHS Trust

Beckett Street, Leeds

LS9 7TF

**Ethics reference:** 23/PR/0461

**You are being invited to take part in a research study.**

Before you decide whether to take part it is important for you to understand why the research is being done and what it will involve. Please take time to read the following information carefully. Part 1 tells you about the study and what is involved. Part 2 tells you how we will use and protect your personal data. Please ask us if there is anything that is not clear or if you would like more information. Take time to decide whether you wish to take part.

**PART 1**

**What is the purpose of the study?**

We want to understand whether having multiple myeloma and treatment for myeloma has an impact on the way the brain processes and uses information (we call this cognitive function).

**Why have I been invited?**

You have been invited to take part because you have accompanied someone to clinic today who has a diagnosis of multiple myeloma. Cognitive function can change as we get older so we need to test both people with and without multiple myeloma of a similar age so that we can tell whether any specific changes occur due to multiple myeloma. We are inviting you to take part because you do not have myeloma.

Subjects who wish to take part in this study will be asked whether they are known to have any conditions which might affect the results (for example conditions such as Alzheimer’s disease or Parkinson’s disease); if you wish to proceed the study team will complete a checklist with you before starting to ensure you are suitable to take part.

**Do I have to take part?**

No. It is up to you to decide whether to take part. If you do, you will be given this information sheet to keep and be asked to sign a consent form. You are still free to withdraw at any time during the duration of the study and without giving a reason. A decision to withdraw at any time, or a decision not to take part, will not affect the standard of care your friend or relative receives. If you chose to withdraw during the study we will use the data we have collected up until the point that you withdraw but we will not collect any further information about you.

**What do I have to do?**

If you agree to take part you will be asked to sign a consent form. We will then ask you to complete a short questionnaire about your physical and mental health, and day-to-day activities. This is to help us to compare these factors between people with and people without myeloma, in case they effect cognitive function. You will not receive any medical assessment, treatment or advice as a result of taking part in this study. This will take around 5-10 minutes.

We will then ask you to complete a set of tests on a computer, which will measure the way your brain processes and uses information and will take approximately 30 minutes. Some people who take part will be asked to wear a head cap containing some light emitting diodes (LEDs) and detectors while they perform these tasks. This won’t restrict the movement of your head or body and shouldn’t be uncomfortable. This will measure how blood and oxygen flow to different part of the brain as you process and use information.

If you attend the clinic again during the time the study is running you will be not be invited to complete the cognitive tests again. This is mentioned here only because patients with myeloma who are taking part in the study may be asked to repeat the cognitive tests at several visits, but as a control participant you will only be asked to do the tests once.

**What are the possible benefits of taking part?**

The information we collect might help improve the treatment and monitoring of people with multiple myeloma.

**What are the possible disadvantages of taking part?**

The risks from taking part in this study are minimal, but it will require some of your time, for which we are very grateful. We will try to ensure the cognitive tests are done whilst you are already waiting in clinic whenever possible. All study activities are covered by the NHS indemnity scheme.

**Are there any restrictions on who can take part?**

You must be over 18 years of age to take part. If you have an existing health condition that affects cognitive function, such as Alzheimer’s disease, you will not be able to take part. Please discuss this with the research team if you are unsure.

**What will happen to any samples I give?**

We will not be collecting any samples from you during this study.

**What will happen to the results of this study?**

The results will be published in a university report and may be reported in peer-reviewed journals and presented at meetings. You will not be identified in any reports.

**Who is organising the study?**

The study is being organised by Dr Christopher Parrish from the Department of Haematology at Leeds Teaching Hospital and Dr Melanie Burke from the School of Psychology at the University of Leeds. The cognitive tests will be run by Miss Sumayyah Patel, who is a PhD student from the University of Leeds and the results will form part of her PhD thesis.

**Who has reviewed the study?**

All research in the NHS is looked at by an independent group of people called a Research Ethics Committee. A favourable ethical opinion has been obtained [REC: 23/PR/0461; IRAS ID: 325678; Version 14.0].

**Who should I contact if I have a complaint?**

If you wish to make a complaint about this study or have been harmed as a consequence of this study please contact the PALS service on 0113 2066261. textphone: 07468753025 (if you are D/deaf or speech impaired) email: patientexperience.leedsth@nhs.net.

**PART 2**

**How will we use information about you?**

We will need to use information from you for this research project.  People will use this information to do the research or to check records to make sure that the research is being done properly. People who do not need to know who you are will not be able to see your name or contact details. Your data will have a code number instead. We will keep all information about you safe and secure.

Once we have finished the study, we will keep some of the data so we can check the results. We will write our reports in a way that no-one can work out that you took part in the study.

**What are your choices about how your information is used?**

- You can stop being part of the study at any time, without giving a reason, but we will keep information about you that we already have.

- We need to manage your records in specific ways for the research to be reliable. This means that we won’t be able to let you see or change the data we hold about you.

**Where can you find out more about how your information is used?**

You can find out more about how we use your information

- at [www.hra.nhs.uk/information-about-patients/](https://www.hra.nhs.uk/information-about-patients/)
- our information on our website <https://www.leedsth.nhs.uk/patients-visitors/patient-and-visitor-information/how-we-use-your-data/>
- by asking one of the research team
- by sending an email to [Leedsth-tr.informationgovernance@nhs.net](mailto:Leedsth-tr.informationgovernance@nhs.net)
- by ringing us on 0113 2433144 and ask for the Data Protection Officer

**IRAS ID: 325678**

**Centre:** Leeds Teaching Hospital NHS Trust

**Participant Identification Number for this trial:**

**CONSENT FORM**

**Title of Project:** Characterising cognitive function in multiple myeloma

**Name of Researcher:** Dr Christopher Parrish

Please initial box

7. I confirm that I have read the information sheet dated.................... (version............) for the above study. I have had the opportunity to consider the information, ask questions and have had these answered satisfactorily.
8. I understand that my participation is voluntary and that I am free to withdraw at any time without giving any reason, without my medical care or legal rights being affected.
9. I understand that data collected during the study may be looked at by individuals from regulatory authorities or from the NHS Trust, where it is relevant to my taking part in this research. I give permission for these individuals to have access to my data.
10. I understand that the information collected about me, and any leftover blood samples may be used to support other research in the future, and that information may be shared anonymously with other researchers.
11. I agree to take part in the above study.

Name of Participant	Date	Signature

Name of Person	Date	Signature

taking consent

1 copy to be retained by the research team within the study site file

1 copy to be provided to the participant

**SUPPLEMENTARY INFORMATION 2A**

Self-reported measures:

i. Patient Health Questionnaire-9 (PHQ-9),

The PHQ-9 is a 9-item self-report screening tool assessing the presence and severity of depressive symptoms over the past two weeks. Each item is rated on a 4-point Likert scale (0 = *not at all* to 3 = *nearly every day*), yielding a total score ranging from 0 to 27.

Higher scores indicate greater depressive symptom severity, with commonly used thresholds:

- 0–4: Minimal
- 5–9: Mild
- 10–14: Moderate
- 15–19: Moderately severe
- 20–27: Severe

The PHQ-9 is widely used in both clinical and research settings and demonstrates strong reliability and validity across populations.

ii. Generalised Anxiety Disorder assessment-7 (GAD-7)

The GAD-7 is a brief, psychometrically robust measure with established validity in both clinical and general populations. It is a 7-item self-report measure assessing anxiety symptoms over the past two weeks. Items are rated on a 4-point scale (0 = *not at all* to 3 = *nearly every day*), producing a total score between 0 and 21.

Severity thresholds are typically interpreted as:

- 0–4: Minimal
- 5–9: Mild
- 10–14: Moderate
- 15–21: Severe

iii. Instrumental Activities of Daily Living (IADL)
     a. Lawton-Brody’s Instrumental Activities of Daily Living

The Lawton–Brody IADL scale assesses more complex functional abilities required for independent living, including telephone use, shopping, food preparation, housekeeping, laundry, transportation, medication management, and financial handling.

Each domain is scored dichotomously (independent = 1, dependent = 0), with higher scores indicating greater functional independence. Total scores range from 0–8 for women and 0–5 for men (to account for historical role differences in scale development).

b. Katz Index of Independence

The Katz Index evaluates basic functional abilities across six domains: bathing, dressing, toileting, transferring, continence, and feeding. Each activity is scored as 1 (independent) or 0 (dependent). Total scores range from 0 to 6, with higher scores indicating greater independence.

**SUPPLEMENTARY INFORMATION 2B**

Each cognitive task was digitalised and hosted on Gorilla Experimental Builder.

i. Corsi Block-Tapping Test (CBTT)

An online version of CBTT was used to as a measure of visuospatial working memory and spatial attention. When administered, the task begins with a fixation cross (0.9 x 0.9cm) presented on-screen for 500ms followed by a 250ms pause. The screen then advances to present nine identical 2.7 x 2.7 cm grey blocks which flash sequentially for 100 ms each. Once the sequence is complete, participants were required to click on the five locations in the order in which they were highlighted. There was a total of 7 trials completed. One practice trial was provided.

ii. Cued Task-Switching

A digitalised version of the Cued Task-Switching test was employed as a measure of cognitive flexibility. Participants engaged in two tasks on a trial-by-trial basis where they either focused upon the properties of colour or shape. Prior to commencing with the task, participants were provided with task instructions followed by a 3000ms blank screen and two practice trials. Each trial started with a fixation cross lasting 250ms before being presented a cue (either the word ‘shape’ or ‘colour’) for 500ms. The target stimulus then appeared, and participants responded with either the F key (corresponding to colour ‘blue’ or shape ‘square’) or J key (for the colour ‘green’ or shape ‘rectangle’). Response feedback would then be provided as a thumbs up or thumbs down icon for 200ms. A total of 16 trials were completed.

iii. Levels of Processing

An adaptation of the classic levels of processing model was used to assess depth of processing involved in memory. The task involved processing of information structurally (by the way it appears), phonemically (by how they sound), or semantically (by associating it with emotions, ideas, or previous knowledge). A fixation cross appeared onscreen for 500ms followed by a question and item which appeared for 2000ms and 1000ms respectively. Participants reacted by clicking ‘Yes’ or ‘No’ in response. Following 30 trials, participants were asked to type in as many items as they could recall. Accuracy scores were based on percentage of correct Yes/No responses.

iv. Posner Cueing Task

A simplified version of the Posner Cueing task was used as a measure of attention. At the start of a trial, participants viewed two boxes (0.9 x 0.9cm) placed on either side of the fixation point. A green thumbs up (stimulus) would appear within either one of the two boxes. Participants were instructed to press the ‘F’ key if the stimulus appeared in the left box and ‘J’ when on the right. There were two practice trials. In 75% of trials, a green circle (cue) would appear before the target stimulus either congruently (where the cues were in the same location as the where the target stimulus is to appear) or incongruently (where cues appear in the opposite location to target). Participants were instructed to not respond to the non-target stimulus. A total of 20 trials were completed.

v. Stop-Signal Delay Task

In this variation of the classic go/no-go task, the task begins with a white circle and fixation cross presented for 1000ms each. The screen then advances to include an arrow stimulus pointing either to the right or left. Participants must respond by pressing the ‘F’ or ‘J’ key to acknowledge whether the arrow is pointing to the left or right. In go trials, the green arrow appears, and participants must respond accordingly. If there is no response within 500 ms, an error message was shown. In no-go trials, the white circle surrounding the arrow turns red for 100 ms prior to the arrow appearing and participants must inhibit their response. Two practice trials were provided.

**SUPPLEMENTARY INFORMATION 3**

**Bayesian modelling used to confirm group differences in cognitive performance.**

**Reaction Time (RT)**

As shown in Figure S1, posterior distributions of group effects on reaction time (RT; ms) were tightly centred around zero across all cognitive domains. Symmetric 95% credible intervals spanned zero and posterior probabilities were close to 0.5 in both directions, indicating strong uncertainty around the direction of group differences. Overall, these results provide no evidence for systematic or domain-specific RT differences between Myeloma participants and controls.

***Figure S1.*** Posterior distributions of group effects on reaction time (RT; ms) across eight cognitive domains: Levels of Processing (LoP_RT), Structural_RT, Semantic_RT, Phonemic_RT, Working Memory (WM_RT), Attention (Attn_RT), Inhibition (Inhib_RT), and Cognitive Flexibility (Flex_RT). Negative values indicate faster RTs in the Myeloma group relative to controls; positive values indicate faster RTs in the Control group. The vertical dashed line represents a null group difference (zero effect).

***Table S1.*** Posterior Summaries for group effects on RT differences (ms) across cognitive domains

| **Cognitive Domain** | **Mean** | **SD** | **95% CrI** | **P(>0)** | **P(<0)** |
| --- | --- | --- | --- | --- | --- |
| LoP | 0.00478 | 0.497 | (-0.969, 0.991) | 0.507 | 0.493 |
| Structural | -0.00522 | 0.492 | (-0.964, 0.955) | 0.498 | 0.502 |
| Semantic | -0.00152 | 0.487 | (-0.958, 0.944) | 0.499 | 0.501 |
| Phonemic | -0.00212 | 0.506 | (-0.992, 0.974) | 0.498 | 0.501 |
| Working Memory | 0.00443 | 0.510 | (-0.992, 1.020) | 0.504 | 0.496 |
| Attention | -0.00591 | 0.505 | (-0.998, 0.969) | 0.493 | 0.507 |
| Inhibition | -0.00379 | 0.501 | (-0.996, 0.987) | 0.502 | 0.498 |
| Flexibility | -0.00752 | 0.502 | (-0.987, 0.964) | 0.489 | 0.511 |

All models demonstrated excellent convergence (R̂ = 1.00), high effective sample sizes, and no divergent transitions or treedepth saturation, indicating stable and reliable posterior estimation (Table S2). Posterior predictive checks indicated good correspondence between observed and model-predicted RT distributions across tasks.

***Table S2.*** Convergence and sampler diagnostics for Bayesian RT models across cognitive domains.

| **Cognitive Domain** | **Max R̂** | **Min Bulk ESS** | **Min Tail ESS** | **Divergent Transitions** | **Treedepth Hits** |
| --- | --- | --- | --- | --- | --- |
| LoP | 1.00 | 16002 | 5178 | 0 | 0 |
| Structural | 1.00 | 16031 | 5726 | 0 | 0 |
| Semantic | 1.00 | 17775 | 5399 | 0 | 0 |
| Phonemic | 1.00 | 17472 | 5607 | 0 | 0 |
| Working Memory | 1.00 | 16913 | 5337 | 0 | 0 |
| Attention | 1.00 | 17887 | 5727 | 0 | 0 |
| Inhibition | 1.00 | 18405 | 5344 | 0 | 0 |
| Flexibility | 1.00 | 13973 | 5505 | 0 | 0 |

*Note: LoP = Levels of Processing.*

All models demonstrated excellent convergence (R̂ = 1.00), high effective sample sizes, and no divergent transitions or treedepth saturation, indicating stable and reliable posterior estimation. Posterior predictive checks indicated good correspondence between observed and model-predicted RT distributions across tasks.

**Accuracy**

As shown in Figure S2, posterior distributions across cognitive domains exhibited a small positive shift favouring the Myeloma group, with posterior means above zero in all tasks. However, 95% credible intervals overlapped zero for every domain, indicating substantial uncertainty around effect magnitude. Posterior probabilities that the group effect exceeded zero ranged from 0.75 to 0.86 (Table S3), suggesting weak-to-moderate directional evidence rather than reliable domain-specific accuracy differences.

***Figure S2.*** Posterior distributions of group effects on task accuracy (logit-transformed) across eight cognitive domains: Levels of Processing (LoP_Correct), Structural_Correct, Semantic_Correct, Phonemic_Correct, Working Memory (WM_Correct), Attention (Attn_Correct), Inhibition (Inhib_Correct), and Cognitive Flexibility (Flex_Correct). Positive values indicate higher accuracy in the Myeloma group relative to controls; negative values indicate higher accuracy in the Control group. The vertical dashed line represents a null group difference (zero effect).

***Table S3.*** Posterior summaries for group effects on accuracy (logit scale) across cognitive domains.

| **Cognitive Domain** | **Mean** | **SD** | **95% CrI** | **P(>0)** | **P(<0)** |
| --- | --- | --- | --- | --- | --- |
| LoP | 0.0976 | 0.139 | (-0.176, 0.364) | 0.756 | 0.244 |
| Structural | 0.0969 | 0.135 | (-0.172, 0.367) | 0.772 | 0.228 |
| Semantic | 0.122 | 0.141 | (-0.149, 0.401) | 0.805 | 0.195 |
| Phonemic | 0.0951 | 0.141 | (-0.185, 0.373) | 0.754 | 0.246 |
| Working Memory | 0.121 | 0.152 | (-0.190, 0.406) | 0.784 | 0.216 |
| Attention | 0.143 | 0.141 | (-0.132, 0.435) | 0.850 | 0.150 |
| Inhibition | 0.104 | 0.135 | (-0.160, 0.373) | 0.780 | 0.220 |
| Flexibility | 0.156 | 0.142 | (-0.128, 0.430) | 0.864 | 0.136 |

Most models demonstrated acceptable convergence (R̂ ≤ 1.03) and adequate effective sample sizes. Lower bulk effective sample sizes were observed for some domains, particularly Working Memory and Attention, reflecting increased posterior uncertainty rather than model instability (Table S4). Posterior predictive checks indicated reasonable correspondence between observed and model-predicted accuracy distributions across domains, with no consistent evidence of model misfit.

***Table S4.*** Convergence and sampler diagnostics for Bayesian accuracy models across cognitive domains.

| **Cognitive Domain** | **Max R̂** | **Min Bulk ESS** | **Min Tail ESS** |
| --- | --- | --- | --- |
| LoP | 1.01 | 885 | 1267 |
| Structural | 1.01 | 790 | 1815 |
| Semantic | 1.00 | 1948 | 1185 |
| Phonemic | 1.00 | 1449 | 738 |
| Working Memory | 1.03 | 109 | 1285 |
| Attention | 1.02 | 664 | 198 |
| Inhibition | 1.00 | 1223 | 887 |
| Flexibility | 1.00 | 3647 | 4386 |

*Note: LoP = Levels of Processing*

**SUPPLEMENTARY INFORMATION 4**

**Mean Reaction Time (RT) x Age**

Across cognitive domains, RTs typically increased with age, although the strength and direction of these associations varied across tasks. These patterns are descriptive and reflect raw unadjusted data. Scatterplots provide a visual representation of unadjusted age–reaction time relationships across domains (Figures S3). To quantify these associations, Pearson correlations were computed for each domain and group (Table S5).

**Table S5.** Age–Reaction Time correlations by group and cognitive domain

| **Group** | **Cognitive Domain** | ***N*** | ***r*** | ***p value*** | **Sig.** |
| --- | --- | --- | --- | --- | --- |
| Control | Attention | 33 | 0.317 | 0.0723 |  |
| Control | Flexibility | 33 | 0.007 | 0.9700 |  |
| Control | Inhibition | 33 | 0.281 | 0.1130 |  |
| Control | LoP | 33 | 0.177 | 0.3250 |  |
| Control | Phonemic | 33 | -0.587 | 0.0003 | ** |
| Control | Semantic | 33 | 0.245 | 0.1700 |  |
| Control | Structural | 33 | 0.108 | 0.5480 |  |
| Control | WM | 33 | 0.290 | 0.1020 |  |
| Myeloma | Attention | 40 | 0.437 | 0.0048 | ** |
| Myeloma | Flexibility | 40 | 0.108 | 0.5090 |  |
| Myeloma | Inhibition | 40 | 0.407 | 0.0092 | ** |
| Myeloma | LoP | 40 | 0.329 | 0.0382 | * |
| Myeloma | Phonemic | 40 | 0.146 | 0.3700 |  |
| Myeloma | Semantic | 40 | 0.312 | 0.0503 | * |
| Myeloma | Structural | 40 | 0.424 | 0.0064 | ** |
| Myeloma | WM | 40 | 0.388 | 0.0133 | * |

*Note:* Correlations represent Pearson’s r between age and log-transformed reaction time within each group and cognitive domain. Significance: * p < .05, ** p < .01. LoP = Levels of Processing; WM = Working Memory.

**Figure S3.** Scatter plots of raw reaction times (RT) against age by group and cognitive domain.

After adjusting for ANCOVA covariates, model-based estimates of RT were derived. Figure S4 presents these model-predicted RTs across group, adjusted for covariates and displayed with 95% confidence intervals. This provides model-based estimates of reaction time across age by group, adjusted for covariates and derived from the fitted ANCOVA model.

**Figure S4.** Model-predicted reaction times by age for myeloma and control participants, adjusted for covariates (age, years of education, and cognitive domain effects).

**Mean Reaction Time (RT) x Years of Education**

In the unadjusted data, there appeared to be no correlation between years of education and RTs in myeloma and control groups (Figure S5). After adjusting for group and covariates (Figure S6), the expected pattern emerged: greater education was associated with faster reaction times across cognitive domains. The myeloma group showed consistently slower reaction times than controls across the education range, but both groups benefited from higher education.

**Figure S5.** Scatter plots of raw reaction times (RT) against years of education by group and cognitive domain.

*Note, 12 (5 myeloma, 7 controls) participants did not report their level of education and were excluded from these visualisations.*

**Figure S6.** Model-predicted RT across years of education by group (Myeloma, Controls).

**Accuracy x Age**

Across all eight cognitive domains, accuracy showed generally weak associations with age in both myeloma and control groups. These patterns are descriptive and reflect unadjusted data. Scatterplots provide a visual representation of raw age–accuracy relationships across domains (Figure S7). To quantify these associations, Pearson correlations were computed for each domain and group (Table S6).

**Table S6.** Age–Accuracy correlations by group and cognitive domain

| **Group** | **Cognitive Domain** | ***N*** | ***r*** | ***p value*** | **Sig.** |
| --- | --- | --- | --- | --- | --- |
| Control | Attention | 33 | -0.127 | 0.4800 |  |
| Control | Flexibility | 33 | -0.047 | 0.7930 |  |
| Control | Inhibition | 33 | -0.072 | 0.6900 |  |
| Control | LoP | 33 | 0.013 | 0.9420 |  |
| Control | Phonemic | 33 | 0.038 | 0.8340 |  |
| Control | Semantic | 33 | 0.055 | 0.7600 |  |
| Control | Structural | 33 | -0.176 | 0.3280 |  |
| Control | Working Memory | 33 | -0.173 | 0.3360 |  |
| Myeloma | Attention | 40 | -0.275 | 0.0856 |  |
| Myeloma | Flexibility | 40 | -0.212 | 0.1890 |  |
| Myeloma | Inhibition | 40 | -0.233 | 0.1470 |  |
| Myeloma | LoP | 40 | -0.287 | 0.0723 |  |
| Myeloma | Phonemic | 40 | -0.258 | 0.1080 |  |
| Myeloma | Semantic | 40 | -0.271 | 0.0904 |  |
| Myeloma | Structural | 40 | -0.165 | 0.3100 |  |
| Myeloma | WM | 40 | -0.079 | 0.6280 |  |

Note: Correlations represent Pearson’s r between age and logit-transformed accuracy within each group and cognitive domain. Significance: * p < .05, ** p < .01. LoP = Levels of Processing; WM = Working Memory.

**Figure S7.** Scatter plots of raw accuracy scores (%) against age by group and cognitive domain.

Model-based estimates derived from the ANCOVA framework (adjusting for group, cognitive domain, age, and years of education) are presented in Figure S8. These indicate generally modest age-related differences in accuracy, with some variation across groups and domains. Controls showed slightly higher accuracy than the myeloma group across the age range, although overall differences were small.

**Figure S8.** Model-predicted accuracy scores by age for myeloma and control participants.

**Accuracy x Years of Education**

In the unadjusted data, raw accuracy scores appeared generally similar between myeloma patients and controls across cognitive domains, with some variability across individuals (Figure S9). After accounting for covariates, model-predicted accuracy was as expected: greater education was associated with higher percentage accuracy (Figure S10).

**Figure S9.** Scatter plots of raw accuracy scores (%) against years of education by group and cognitive domain.

*Note, 12 (5 myeloma, 7 controls) participants did not report their level of education and were excluded from these visualisations.*

**Figure S10.** Model-predicted accuracy across years of education by group (Myeloma, Controls).

**SUPPLEMENTARY INFORMATION 4**

**Treatment x MoCA Scores in Myeloma Group**

Within the MM group, certain treatment-related factors—specifically steroid use and a history of haematopoietic stem cell transplant (HSCT)—were associated with slightly higher MoCA scores. Mean MoCA scores were approximately 25.7 among steroid users and 25.9 among HSCT recipients, compared with around 24.8 for non-users. Median values followed a similar pattern (26–27 vs 25), with HSCT recipients showing the highest median score (27), suggesting a modest association with preserved cognitive function. Non-users and non-HSCT patients, by contrast, had a greater proportion of lower scores (<25). Although descriptive, these findings indicate that certain treatment exposures may be linked to relatively better cognitive outcomes within the MM cohort, potentially reflecting treatment-associated variability across regimens. Mean MoCA scores by treatment type are shown in Figure S11.

**Figure S11.** Distribution of Montreal Cognitive Assessment (MoCA) scores in multiple myeloma (MM) patients by treatment status. (A) Steroid use. (B) Haematopoietic stem cell transplant (HSCT).
